# *Legionella pneumophila* Infections during a 7-Year Retrospective Analysis (2016–2022): Epidemiological, Clinical Features and Outcomes in Patients with Legionnaires’ Disease

**DOI:** 10.3390/microorganisms11020498

**Published:** 2023-02-16

**Authors:** Tommaso Lupia, Silvia Corcione, Nour Shbaklo, Barbara Rizzello, Ilaria De Benedetto, Erika Concialdi, Anna Sara Navazio, Maurizio Penna, Maria Teresa Brusa, Francesco Giuseppe De Rosa

**Affiliations:** 1 Unit of Infectious Diseases, Cardinal Massaia, 14100 Asti, Italy; 2 Department of Medical Sciences, Infectious Diseases, University of Turin, 10126 Turin, Italy; 3 School of Medicine, Tufts University, Boston, MA 02111, USA; 4 Microbiology Unit, Cardinal Massaia Hospital, 14100 Asti, Italy

**Keywords:** *Legionella*, Legionnaires disease, pneumonia, urinary antigen, neutrophil-to-lymphocyte ratio, lymphopenia

## Abstract

*Legionella pneumophila* (LP) is one of the main causative agents of community-acquired pneumonia in Europe and its fifth bacterial cause in Italy (4.9%). We conducted a seven year retrospective analysis of LP infection serogroup 1 in Asti, Piedmont, between 2016 and 2022. Patients were included if they tested positive for the *Legionella* urinary antigen. Clinical, laboratory, and radiologic data were analyzed to describe the risk factors for mortality. Fifty patients with LD were collected, mainly male, with a median age of 69 years. The main comorbidities were cardiovascular diseases (50%), pulmonary diseases (26%), and neurological diseases (12%). The most common clinical presentations were fever, respiratory, gastrointestinal, and neurologic symptoms. Older age (*p* = 0.004), underlying cardiovascular diseases (*p* = 0.009), late diagnosis at admission (*p* = 0.035), and neurological symptoms at diagnosis (*p* = 0.046) were more common in the non-survivor group. Moreover, a septic-shock presentation or the need for non-invasive ventilation at admission were associated with a higher mortality. No considerable differences in the biochemical data were found between the two groups except for the median neutrophil count, lymphocyte count, neutrophil-to-lymphocyte ratio, and PCT value. We did not find any differences in mortality related to the choice of antibiotic regimen. Differences in outcome were associated with the median duration of treatment (*p* =< 0.001) but not to the choice of antibiotic regimen (mainly levofloxacin or azithromycin). In conclusion, early individuation of the wide spectrum of clinical characteristics of LP infection such as respiratory, cardiac, and neurological manifestations of the patient’s comorbidities, and significant biochemical data should help clinicians flag high risk patients and potentially improve their outcome.

## 1. Introduction

*Legionella pneumophila* (LP) infection was first recognized in an outbreak in the summer of 1976 during the annual American Legion three-day convention at the Bellevue-Stratford Hotel in Philadelphia, Pennsylvania [1]. During this first local contagion, a total of 182 cases were reported, and 29 people died; most of the patients complained of tiredness, chest pains, lung congestion, and fever [1].

In December 1976, microbiologists Joseph McDade and Charles C. Shepard discovered LP after observing that guinea pigs became ill after being inoculated with lung tissues from individuals who had passed away from LP [2]. It was found that these bacteria were the causative organism of Legionnaires’ disease (LD), and they were subsequently given the name *Legionella* (for Legionnaires) *pneumophila* (from the Greek words *pneumon* [lung] and *philos* [loving]) [3,4].

LP is now one of the main causative agents of community-acquired pneumonia (CAP) in Europe. Globally, LP serogroup 1 is the leading cause of LD, accounting for 90% of diagnoses [5]. According to Welte and colleagues, LP infection is the fifth bacterial cause of CAP in Italy (4.9% of patients), as reported in their recent epidemiological European scenario [5].

**Figure 1:**
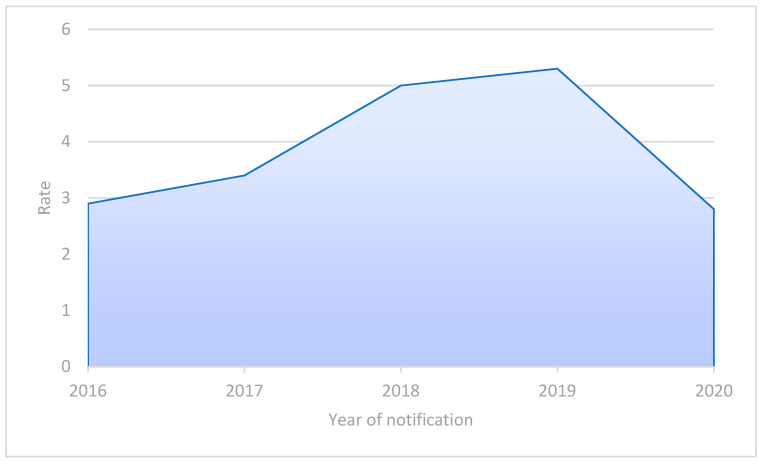
The LD rates per 100,000 population in Italy per year, 2016–2020, modified from [6].

In the most recent annual report for 2020, which was published in May 2022, the ECDC stated that the total notification rate for LD was 1.9 cases per 100,000 people in the European Union (EU) (Figure 1). However, LD is likely underdiagnosed, so this data may underestimate the true incidence. Of the reported cases, 75–80% were over 50 years and 60–70% were male [6]. In 2021, the LD incidence in Italy was 4.6 cases/100,000, with a median age of 67 years. A total of 84% was classified as community-acquired infections [7]. Moreover, in 2020, 19 community- or hospital-acquired outbreaks were reported across six European countries (Germany, Italy, The Netherlands, Norway, Portugal, and Spain), where the majority (4866; 58%) of cases occurred between June and October, according to the classically described seasonality in Europe [6].

Using oropharyngeal swabs for culture and PCR, paired blood samples for serology, and urine for *Legionella* antigen detection, a well-cited research by Arnold and colleagues of 4337 patients from 21 countries reported a global incidence of 22%, with minor changes between continents [8].

In its natural environment, the risk of human LP infection is rare, but the risk increases when bacteria are aerosolized. Inhalation or aspiration of LP bacteria from the environment can lead to the development of LD. In fact, *Legionella* spp. bacteria can be discovered naturally in habitats containing freshwater such as lakes and streams. They are also able to cultivate and propagate within the water systems of man-made buildings (i.e., showerheads and sink faucets, cooling towers, hot tubs, decorative fountains and water features, hot water tanks and heaters, plumbing systems, and windshield wiper fluid tanks) [8]. Conversely, air conditioning systems in homes and vehicles do not pose a concern for the spread of *Legionella*, as long as they do not use water to chill the air [9,10].

The optimal growth temperature for LP is 35 °C, but the bacteria can thrive at ranges between 25 and 42 °C [11]. Humans are responsible for creating most legionellosis hotspots, which are artificial bodies of water with temperatures significantly higher than the surrounding air [11]. The human impact on the environment is mostly responsible for the emergence of legionellosis in the latter part of the 20th century. Natural freshwater settings have not been linked to reservoirs of legionellosis outbreaks, suggesting that, if left alone, *Legionellae* would be a very infrequent cause of human disease [11].

In most cases, LD is not transmitted from one person to another [6,9]. In 2016, Correia and colleagues [12] reported one of the first probable cases of human-to-human transmission of *Legionella* spp. It occurred theoretically in a small non-ventilated room from an LP-positive woman caring for her severely ill son, who became infected in the days after by the same *Legionella* genotype bacteria.

This is the first seven-year retrospective analysis from the Province of Asti in the Piedmont region for the 2016–2022 period. Interestingly, Riccò and colleagues described the epidemiology of LD in Italy between 2004 and 2019, reporting a total of 23,554 LD cases during the study period [13]. Among them, 1549 cases (6.6%) were from Piedmont, which was the sixth-highest region for the incidence of LP cases, with an average-year incident case number of 95.4 (range 79.6–111.1) [13].

In this retrospective analysis, we aimed to describe the epidemiology of LD and its clinical characteristics, notable clinical complications, and outcomes by using data from a seven-year period in the Province of Asti in the Piedmont region (Italy).

## 2. Materials and Methods

This retrospective analysis was conducted in the Province of Asti, Cardinal Massaia Hospital, a tertiary care hospital in the Piedmont region of Italy.

Patients were included if they were positive for LP serotype 1 in urinary antigen testing (UAT) in the emergency department and/or ≤48 h from hospital ward admission for community-acquired infections or ≥48 for hospital-acquired infections and complained of characteristics of LD, according to Cunha et al. [14].

Data were collected from electronic medical records between 2016 and 2022 and included information on the timing of the disease, signs and symptoms, imaging, and laboratory results on admission. Pneumonia was diagnosed based on radiologic abnormalities (i.e., pulmonary infiltrates, pulmonary consolidations, and ground-glass opacities) by chest X-rays. The laboratory assessments comprised a complete blood count, blood chemical analysis, coagulation testing, liver and renal function assessment, C-reactive protein (CRP), procalcitonin, lactate dehydrogenase (LDH), ferritin, sodium, potassium, and creatinine tests at admission. Laboratory testing comprised samples taken at the baseline. Fever was defined as a temperature >37.5 °C. The LP urinary antigen was detected with an automated qualitative immunofluorescence assay test (UAT; Sofia Legionella FIA Kit, Quidel, San Diego, CA, USA). The results were displayed as positive or negative. Sepsis and septic shock were defined according to SEPSIS-3 [15].

The need for informed consent was waived due to the study’s retrospective nature, which the medical direction of the hospital approved (N. Prot. CE 0031285). Data were collected according to Italian laws on privacy.

### Statistical Analysis

Descriptive data were reported in frequency and percentage for the categorical variables and in the median and range for the continuous variables. Yearly trends for LD are shown across the years. Categorical variables were compared to mortality using the Chi-squared test, and continuous variables were compared to mortality using the *t*-test. All analyses were performed using SPSS v. 27.0 (SPSS Inc., Armonk, NY, USA), and the two-tailed statistical significance was set at <0.05.

## 3. Results

### 3.1. Patient Characteristics

We collected a total of 50 patients with a diagnosis of LD over the study period (Table 1).

**Figure 2:**
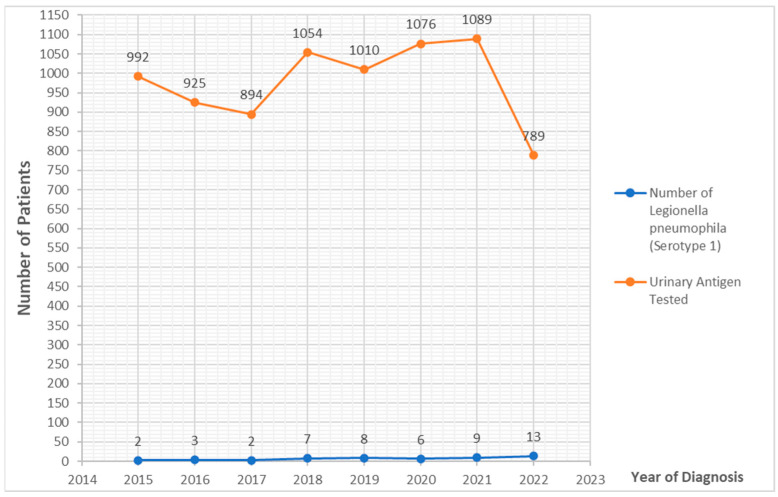
Trend of LD toward years of study.

All diagnoses were defined by the detection of the *Legionella pneumophila* serovar 1 UAT, resulting in a median of six cases (range 2–14) reported every year between 2015 and 2022 (Figure 2).

Ninety-eight percent of patients were diagnosed with a community-acquired LD. The overall mortality, considering the outcome at 7 and 28 days, was 12% (six patients out of 50). The ward of admission was mainly the medical ward in 94% of cases. We compared the demographic, clinical, laboratory, and radiological features between the two groups, alive and dead patients, to define the risk factors for mortality. The median age among our patients was 69 years (range 34–92); 80% (N = 40/50) of them were male and 98% were Caucasian (49/50). Through physiological anamnesis, 34% were active smokers (17/50, data not available from 29 of them) and 6% were daily alcohol drinkers (3/50, data not available from 44 of them). The main underlying diseases and baseline characteristics of our population were cardiovascular diseases (50%), pulmonary diseases (26%), and neurological diseases (12%). Twenty-four percent were immunosuppressed due to HIV or oncological/hematological diseases. Among the patient population features, older median age and the presence of cardiovascular comorbidities resulted in statistically significant risk factors related to the non-survivor group. Diagnosis was ruled in at time of admission in most cases, but when comparing the survivor and non-survivor groups, one day median time to diagnosis was found to be linked to poor prognosis. However, in both groups, an appropriate antibiotic therapy was started at admission.

**Figure 3:**
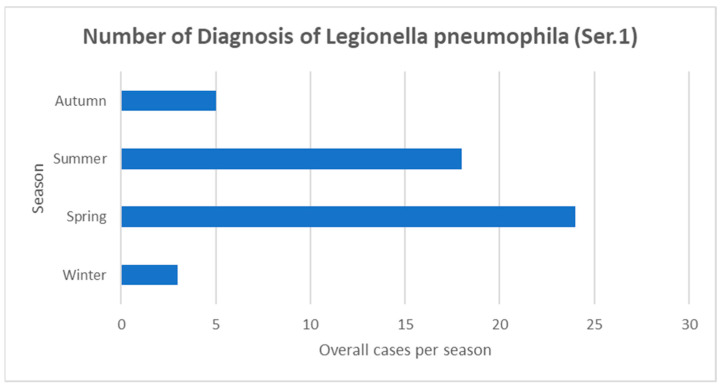
Seasonality of LD.

We also divided cases according to seasonality, as presented in Figure 3, noticing a marked prevalence in warmer seasons, with a peak of incidence in spring.

### 3.2. Clinical Presentation

Clinical manifestations were mainly represented by fever, respiratory symptoms, gastrointestinal symptoms, and neurological symptoms. The constantly observed clinical feature was fever, presented in all fifty patients, who also complained in 90% of cases of respiratory symptoms. Furthermore, 74% of patients developed respiratory failure and needed oxygen support from low flow to non-invasive mechanical ventilation (NIV), as reported in Table 1. Indeed, the presence of respiratory failure requiring NIV showed an association with poor outcome. Moreover, 12% of patients experienced neurological manifestations, with a significantly greater prevalence in the non-survivor group. Among the complications analyzed, the most frequent were pleural effusion (28%), acute kidney injury without rhabdomyolisis (20%), and primary rhabdomyolosis (16%). A total of 10% of patients was diagnosed with a new onset cardiac arrythmia. A few co-infections/superinfections were also encountered such as three cases of BSI (bloodstream infections by *S. hominis*, *S. capitis*) and single cases of *C. difficile* infection, COVID-19 pneumonia, and bacterial pneumonia (*P. aeruginosa*).

### 3.3. Laboratory and Radiological Findings

As shown in Table 2, the laboratory data and radiologic findings at the time of diagnosis were collected.

No considerable differences in the biochemical data were found between the two groups except for the median neutrophil count, lymphocyte count, and the neutrophil-to-lymphocyte ratio: in the non-survivor group, the neutrophil and lymphocyte counts were lower than in the survivor group, but the neutrophil-to-lymphocyte ratio was significantly increased, probably suggesting a more prominent impact of lymphopenia on the outcome. Where available (42% of cases), data on the presence of microhematuria were considered, and it was found that 32% of patients tested positive. The median R-CP levels were 277 mg/dL (normality cut-off 5 mg/dL). All patients underwent chest X-rays at the time of diagnosis: only one patient, belonging to survivors group, was diagnosed with a multifocal pneumonia. The other 48 radiography findings consisted of isolated lung infiltrate, mainly localized in lower and median lobes. Besides, as previously said, 14 patients developed also pleural effusion. All patients received appropriate antibiotic treatment: 45 patients received levofloxacin-based PO/EV (32 patients) or azithromycin-based PO monotherapy (5 patients). The 5 patients left were given combination therapy with levofloxacin + azithromycin (4 patients) or levofloxacin + rifampin (1 patient). No differences on outcome were attributed to the choice of regimen. However, difference on outcome was associated to median duration of treatment, as it can be noticed that survivor vs non-survivor group, it was 14 days vs 10 days.

## 4. Discussion

We reported epidemiological, clinical features and outcomes of 50 cases of LD that were retrospectively collected in Asti, Piedmont, Italy over the course of seven years. In addition, we analyzed the risk factors for mortality in this cohort, pairing data with the available literature regarding LD. Moreover, to our knowledge, original articles regarding the clinical features of LD in Piedmont (Italy) have historically been uncommon [16,17].

In recent years, the majority of cases of LD in Italy and Europe have been in patients older than 50 years old (70.4%) or over 45 years old (91%) [13,18]. We confirmed in this analysis that LD is more common in older adults, as reported in different other studies [18,19,20]. We found a median age of 69 years for patients with LD, while only four patients were younger than 45 (8%). In addition, we found that aged people presented a higher risk of mortality (*p* = 0.004) when they were diagnosed with LD. Age has also been reported as a predictor of mortality in other studies regarding LD or involving patients with CAP [19,20]. There is a very close link between Legionella and age. Due to the peculiarities of its life cycle, LP can escape detection by the immune system of its host [21,22,23]. The higher rate of infections in older adults are likely related to the intrinsic characteristics of this pathogen and probably to acquired immunodeficiency due to ageing. Failure to develop protective humoral and cellular immune responses to a pathogen or vaccination and a systemic low-grade inflammatory state (called “inflammaging”) are both hallmarks of immunosenescence [24].

In our series, we faced a higher rate of male patients (80%) in the overall population. According to available data from a recent Italian systematic review, males are more frequently diagnosed with LD (70.4%) [13]. Furthermore, in the last ECDC report on LD, the overall male-to-female ratio was 2.3:1, with 7.1 cases per 100,000 population in males and 2.8 in females [18]. Interestingly, we found a higher percentage of females in the non-survivor group, and differences between the two groups (survivors versus non-survivors) were near to statistical significance (*p* = 0.081). Recently, Chidiac and colleagues, in one of the most extensive studies to date regarding LD, have shown that female sex to be a predictor of mortality among a cohort of 540 patients (RH 2.00, 95% CI 1.08–3.69) [19].

In addition, we collected retrospective habits of patients, including daily alcohol consumption and smoking. The discussion of these data, however, is undermined by the lack of data for many patients. Thirty-four per cent of patients were active smokers, smoking was more common in survivors who complained of LD and was close to statistical significance (*p* = 0.061). Active smoking is commonly considered a risk factor for community-acquired LD [25]. Tobacco smoking causes morphological changes in the epithelium of the bronchial mucosa, with loss of cilia, mucous gland hypertrophy and increased goblet cells that may favor the presence and spread of microbes in the bronchial tree [26]. Moreover, Strauss and colleagues showed that smoking is the most critical risk factor in LD subjects, as the risk may be increased by 121% for each pack of cigarettes consumed daily, with an Odds Ratio of 3.48 [25]. Although smokers in our retrospective analysis were younger than the non-survivor group with a median age of 57 (range 38–87), this factor is likely related to the lower mortality reported.

Cardiovascular, pulmonary, and neurological diseases were the most frequent comorbidities in the overall population and the non-survivor group. Furthermore, we showed that having a history of cardiovascular disease was linked to high mortality (*p* = 0.009). These findings confirm previously reported data from el-Ebiary and colleagues [27]. In addition, *Legionella* spp., like other atypical pathogens that cause pneumonia, have a propensity to determine cardiac signs or symptoms, for example, producing relative bradycardia in the presence of fever, a sign called Faget’s sign [28]. Finally, Serrano Fernandez et al. showed that developing new cardiovascular events (CVE) during LD hospitalization is by no means an uncommon complication. In their series of 243 patients, 13.6% developed CVE [29].

Moreover, Falcone and colleagues found that new cardiovascular events during LD were associated with a higher risk of ICU admission (OR 10.91, confidence interval 95% 2.83–42.01, *p* = 0.001) and worse outcomes [30]. In our patients, despite the absence of statistical significance, we found a higher incidence of new-onset arrhythmia (33% vs. 6.8%) in non-survivors. Our findings related to cardiovascular comorbidities should be interpreted cautiously because of the high prevalence in the general population, especially in the age groups included in our analysis. However, despite the wide variety of cardiac complications reported in LD patients [29,30] that are linked to unfavorable outcomes, a history of heart or pre-existent vascular disease should be a cause for suspicion in clinicians dealing with more aggressive clinical presentations.

Clinical presentations at admission in our population were characterized by fever in all patients. Other frequent signs and symptoms were respiratory symptoms, the onset of a new pleural effusion, and acute kidney injury (AKI).

Patients with LD and respiratory symptoms frequently needed oxygen support, and subjects requiring NIV (non-invasive ventilation) or C-PAP (continuous positive airway pressure) showed a higher mortality in this series (*p* < 0.001). A previous report found that the use of NIV may delay ICU admission and is probably associated with a poorer outcome [31].

Pleural effusion and AKI were more frequent in the non-survivor group without statistical significance. Several authors have found that the development of pleural effusion during LD infections is linked with a worse prognosis [32,33].

AKI is not uncommon in LD, and the frequency of AKI reported in the literature ranges from 13 to 15% [33].

Interestingly, neurological symptoms were presented in 10% of cases and resulted in a higher mortality rate in our retrospective analysis (*p* < 0.046). Neurological involvement in LD includes encephalitis, meningitis, peripheral nerve disease, and brain stem abnormalities [34]. Patients may present with altered consciousness, hallucinations, delirium, and cerebellar ataxia [35]. LD can manifest with neurological symptoms; however, having an isolated neurological presentation is rare. It is a difficult diagnosis due to its ambiguous clinical presentation, which lacks specific characteristics suggesting LD [36]. These neurological symptoms often lead to an extensive workup, and the complexity of diagnostic workups can significantly influence the patient outcomes [35,36].

We confirmed the previous literature findings that highlighted the higher mortality of patients with a delayed diagnosis of LD (*p* < 0.001) [35,36]. Despite this, however, our retrospective analysis did not find any delayed diagnosis due to neurological symptoms and misdiagnosis. In addition, central nervous system involvement should indirectly define the severity of extrapulmonary manifestations.

Among the laboratory abnormalities, we found a lower count of lymphocytes, a higher amount of neutrophils, and a higher NLR to be associated with higher mortality. Interestingly, in a recent systematic review by Kuikel and colleagues, the association between NLR and adverse outcomes of patients with CAP was evaluated [37]. This association was evaluated in the nine studies included in the review and it was found to be significant in all of them [37]. However, NLR was shown to have a high mortality prediction compared to the neutrophil count or lymphocyte level alone. However, a study carried out by Kaya et al. [38] concluded that NLR is not superior to the commonly used scoring system (PSI, CURB-65) in estimating mortality. Nevertheless, NLR should be used as a rapid tool for flagging high-risk patients in patients admitted for LD.

Furthermore, isolated lymphopenia in patients with symptoms compatible with LD was defined as a marker for earlier definitions of LP CAP despite a high count of neutrophils [39]. Nevertheless, LD is characterized by the accumulation of activated T cells in the lungs and is likely to be the basis of this earlier absolute lymphocytopenia [40]. The role of neutrophils in LP infections seems crucial, although via immunomodulatory effects and not direct killing. In fact, the early accumulation of neutrophils at sites of infection is a consistent observation in Legionella pneumonia in both animal models and humans [41].

We also found that procalcitonin was higher in the non-survivor group (*p* = 0.005). Many authors have highlighted that in LD, procalcitonin levels on admission might be a promising predictor for adverse medical outcomes [42,43].

Herein, we described the seasonality of the presentation of LD. We found a higher incidence of cases in spring, followed by summer. In the recent report by the ECDC of 2020 [6], the distribution of cases by month of reporting shows that the majority (4866; 58%) of cases occurred between June and October, similar to previous years and in line with the known seasonality of LD in Europe, which peaks in summer. In our region, in the years considered, the peak of LD was earlier than expected, according to the European data [6]. Moreover, when we matched the total with positive UAT for LP performed during the period of study, we found a higher number of patients tested in 2020–2021, as shown in Figure 2. This increase in testing was mainly linked to the increase in the admission of patients with respiratory tract infections due to the COVID-19 pandemic. Despite the increase of testing in patients with suspected respiratory disease, the total cases of LP found was substantially stable with respect to 2018–2019. Unfortunately, in most cases, to rule out co-infections or super-infections, testing for LP was performed not only in patients who were highlighted as being suspected of having LD, but in all patients with a known infection due to SARS-CoV-2.

Despite a higher rate of cases of SARS-CoV-2 infections in our region during the years 2020–2022 [44], we found a single case of co-infection between LP and SARS-CoV-2.

In our series, the main test for diagnosis was the urinary antigen test (UAT). Riccò et al. [11], in their systematic review, confirmed the wide utilization of this diagnostic method in Italy, with the test being the first diagnostic test used in 95.3% of cases of LP. As a result of its numerous benefits, the Legionella UAT has quickly become the test of choice for the diagnosis of LD [45]. UATs for Legionella are quick, painless, non-invasive, and easy to carry out. They are also unaffected by past antibiotic use [46]. In particular, Legionella UATs make it possible for individuals suffering from severe legionellosis to receive early and sufficient therapy. Unfortunately, the great majority of Legionella UATs can only detect LP serogroup 1, which is the most common strain of the bacteria. Although LP serogroup 1 is responsible for more than 80–95% of legionellosis cases across the majority of the United States and Europe, other species and serotypes are more prevalent in certain regions such as the southern and Pacific regions of the United States, New Zealand, and Australia. Because there are 58 distinct species of Legionella and over 70 serogroups, the utility of the Legionella UAT diminishes along with the prevalence of serogroup 1, with the result that there are 58 different serogroups.

On the other hand, in 2019, the Asahi Kasei Pharma Corporation in Japan introduced a novel urinary antigen test kit known as Ribotest Legionella [46]. This test kit can identify all serogroups of LP as well as Legionella species other than LP, and so has been marketed under the name Ribotest Legionella. This new UAT can improve the early and appropriate diagnosis of LD due to non-LP serogroup 1, thereby improving prognosis. Although further research is required to evaluate the impact and usefulness of this novel kit in other countries, this test can potentially improve early and appropriate diagnoses. However, due to extended antigen excretion, Legionella UAT also has the potential to provide false-positive results in individuals who have just recovered from LD, which is one of the test’s many drawbacks [46,47].

According to different therapeutic regimens used for our cases of LD, mainly levofloxacin- or azithromycin-based ones, we did not find any differences in mortality. Recently, Jasper and colleagues obtained data in a systematic review that confirmed an absence of difference in the effectiveness of fluoroquinolones versus macrolides in reducing the mortality among patients with LP [48].

On the other hand, we found that non-survivors reported shorter lengths of days of treatment (*p* < 0.001), in line with the literature in this field [49]. These findings are difficult to understand due to the possibility that early death is the main risk for shorter therapeutic regimens.

There are several limitations to this study. This is a single-center study that may not accurately reflect the general demographics of Italy. We did not include LD diagnoses other than the urinary antigens. For this reason, other *Legionella pneumophila* serotypes were not detected, and therefore, LP/LD prevalence might be underestimated. Moreover, various data are missing, in particular, phosphatemia, ferritin levels, blood gas levels, and the computed tomography data. Finally, there was a lack of data on alcohol consumption and smoking for a high number of patients included in the study.

## 5. Conclusions

Considering its essential role among the etiologies of CAP in Europe and Italy, we have acquired more knowledge about LP and its clinical syndromes over recent years. The early definition of anamnestic, clinical, and laboratory risk factors for mortality in patients affected by LD might help alert clinicians to more aggressive presentations. In our retrospective analysis, we focused on older median age, history of cardiovascular comorbidity, neurological symptoms, respiratory failure, and an increased NLR as features linked to a poorer outcome in our cohort. Early diagnosis and awareness is pivotal to reduce the risk of poorer outcomes in LD patients, however, despite this, the use of rapid tests such as UAT should be focused in higher risk patients, and LP infections other than serotype 1 could be missed with these standard urinary tests.

## Figures and Tables

**Table 1:** The main characteristics of hospitalized patients with Legionnaires’ disease.

Main Characteristics of Hospitalized Patients with Legionnaires’ Disease (N = 50) N (%) or Median (Range)		Survivors, N (%) or Median (Range) N = 44 (88)	Non-Survivors, N (%) or Median (Range) N = 6 (12)	*p* Value
Age (median)	69 (range 34–92)	66 (34–92)	85 (79–88)	0.004
Sex	20% Female (10/50); 80% Male (40/50)	16% Female (7/44); 84% Male (37/44)	50% Female (3/6); 30% Male (3/6)	0.086
Ethnicity	98% Caucasian (49/50); 2% Rom	97% Caucasian (43/44); 2.3% Rom (1/44)	100% Caucasian (6/6); 0% Rom	>0.99
Smoking	34% Active smokers (17/50)	38.6% (17/44)	0% (0/6)	0.061
Alcohol	6% Daily Drinkers (3/50)	6.8% (3/44)	0% (0/6)	0.509
Comorbidities				
Cardiovascular diseases	25% (25/50)	43.2% (19/44)	100% (6/6)	0.009
Kidney %	8% (4/50)	9.1% (4/44)	0% (0/6)	0.441
Pulmonary diseases	26% (13/50)	25.6% (11/44)	33.3% (2/6)	0.687
Neurological diseases	12% (6/50)	11.4% (5/44)	16.7% (1/6)	0.708
Solid tumor	10% (5/50)	9.3% (4/44)	16.7% (1/6)	0.577
Hematological disease	8% (4/50)	9.3% (4/44)	0% (0/6)	0.436
Diabetes Mellitus	6% (3/50)	4.5% (2/44)	16.7% (1/6)	0.241
SOT	0% (0/50)	--	--	--
Rheumatologic diseases %	0% (0/50)	--	--	--
HIV	6% (3/50)	6.8% (3/44)	0% (0/6)	0.509
Hepatic diseases	16% (8/50)	12.2% (5/44)	0% (0/6)	0.366
Obesity	14% (7/50)	23% (6/44)	25% (1/6)	>0.99
LD Acquisition Setting and Ward of Admission				
Community-acquired	98% (49/50)	97.7% (43/44)	100% (6/6)	0.709
Hospital-acquired	2% (1/50)	--	--	--
Ward of admission	94% MW; 4% ICU; 2% SW	--	--	--
Diagnosis at Admission				
Urinary antigen for *L. pneumophila* serotype 1	100% (50/50)	--	--	--
Diagnosis from admission (days)	Median 0 (range 0–12)	0 (0–4)	1 (1–9)	0.035
Specific therapy from admission	Median 0 (range 0–10)	0 (0–2)	0 (0–0)	0.899
Clinical Presentations and Complications at Admission				
Fever	100% (50/50)	--	--	--
Respiratory symptoms	90% (45/50)	90.9% (40/44)	83.3% (5/6)	0.562
Gastro-intestinal symptoms	12% (6/50)	13.6% (6/44)	0% (0/6)	0.335
Neurological symptoms	10% (5/50)	7% (3/44)	33.3% (2/6)	0.046
Primary rhabdomiolysis	16% (8/50)	--	--	--
Acute Kidney Injury (without rhabdomiolysis)	20% (10/50)	18.2% (8/44)	33.3% (2/6)	0.384
Pleural Effusion	28% (14/50)	27.3% (12/44)	33.3% (2/6)	0.756
New onset arythmia	10% (5/50)	6.8% (3/44)	33% (2/6)	0.103
Liver inflammation	12% (6/50)	13.6% (6/44)	0	>0.99
Septic shock (according to SEPSIS-3)	2% (1/50)	0	16.7% (1/6))	0.006
Respiratory failure	74% (37/50) *Low Flow Oxygen* (34/50) *NIV/C-PAP* (3/50)	(30/44) (0/44)	(4/6) (3/6)	0.941 <0.001
Co-infections/Superinfections				
BSI	*S. hominis* (2); *S. capitis* (1)			
CDI	*Clostridioides difficile* (1)			
Viral pneumonia	SARS-CoV-2 (1)			
Bacterial pneumonia	*Pseudomonas aeruginosa* (1)			
Outcomes				
Survival 7 days after diagnosis	92% (46/50)			
Survival 28 days after diagnosis	88% (44/50)			

Abbreviations: N: number; SOT: solid organ transplant; HIV: Human Immunodeficiency Virus; BSI: blood-stream infection; CDI: *Clostridioides difficile* infection; NIV: non-invasive ventilation; C-PAP: continuous positive airway pressure; ICU: intensive care unit; LD: Legionnaires’ disease.

**Table 2:** The main laboratory characteristics of hospitalized patients with Legionnaires’ disease.

Main Characteristics of Hospitalized Patients with Legionnaires’ Disease (N = 50) N (%) or Median (Range)		Alive, N (%) or MEDIAN (Range)	Dead, N (%) or Median (Range)	*p* Value
WBC	13,715 (2300–31,210)	12,510 (2300–31,210)	14,130 (11,160–14,560)	0.074
PLTS	191,000 (77,000–702,000)	154,000 (77,000–466,000)	191,000 (140,000–365,000)	0.285
Lymphocytes	810 (170–28,210)	830 (210–28,020)	420 (220–810)	0.005
Monocytes	400 (50–1770)	340 (50–1640)	530 (50–700)	0.271
Eosinophils	40 (0–460)	20 (0–260)	110 (30–130)	0.987
Neutrophils	11,090 (2030–28,850)	9055 (2030–18,510)	114 (8750–13,530)	0.043
Neutrophil-to-Lymphocyte Ratio	11.9 (0.1–89.8)	9.84 (0.10–30.81)	20.83 (14.16–61.5)	<0.001
GOT	44 (15–288)	60.5 (15–288)	92 (37–117)	0.771
GPT	44 (8–228)	46.5 (14–228)	39 (14–53)	0.203
Na	129 (127–152)	134.5 (126–152)	137 (134–143)	0.368
K	3.4 (3.0–4.0)	3.9 (3–5)	3.8 (3–4)	0.261
LDH	528 (335–1098)	547.5 (222–1098)	771 (578–780)	0.301
Ferritin	1634 (344–5810)	1221 (344–5481)	-	-
Creatinine	0.96 (0.42–4.95)	0.89 (0.58–1.48)	1.1 (0.87–1.5)	0.493
C-RP (mg/dL)	277 (29–478)	316 (103–478)	386 (73–432)	0.691
PCT	1.96 (0.12–37)	2 (0.99–7.32)	9.9 (6.6–17.5)	0.005
Microhematuria	*Positive*, 32% (16/50) *Absent*, 10% (5/50) *Not Available*, 58% (29/50)	*Positive*, 73.7% (14/50) *Absent*, 26.3% (5/50)	*Positive*, 100% (2/50) *Absent*, 0% (0/50)	0.406
*Radiographic Involvement*				
Isolated Median Lobe	28% (14/50)	27.3% (12/50)	33.3% (2/50)	0.756
Isolated Apical Lobe	16% (8/50)	13.6% (6/50)	33.3% (2/50)	0.217
Isolated RIL	26% (13/50)	22.7% (10/50)	50% (3/50)	0.153
Isolated LIL	12% (6/50)	13.6% (6/50)	0% (0/50)	0.335
Multifocal Involvement	2% (1/50)	2.3% (1/50)	0% (0/50)	0.709
*Choice of Treatment*				
Levofloxacin (IV/OR)	64% (32/50)	80.6% (29/50)	60% (3/50)	0.567
Levofloxacin (IV/OR) + Azithromicin (OR)	10% (4/50)	8.3% (3/50)	205 (1/50)	
Levofloxacin (IV/OR) + Rifampicin (IV/OR)	2% (1/50)			
Azithromicin (OR)	26% (13/50)	83.3% (10/50)	100% (3/50)	0.749
*Duration of Treatment*				
Days of treatment	14 (1–21)	14 (6–21)	10 (1–14)	<0.001

Abbreviations: IV: intravenous; OR: oral route; LIS: left inferior lobe; RIL: right inferior lobe; PCT: procalcitonin; C-RP: C-reactive protein; LDH: lactate dehydrogenase; GOT: glutamic oxaloacetic transaminase; GPT: glutamic pyruvate transaminase: PLTS: platelets; WBC: white blood cells; N: number.

## Data Availability

The data presented in this study are available on request from the corresponding author.
